# Childhood adversity and late-life depression: moderated mediation model of stress and social support

**DOI:** 10.3389/fpsyt.2023.1183884

**Published:** 2023-06-26

**Authors:** Jin-kyung Lee, Jinhee Lee, Moo-Kwon Chung, Ji Young Park, Taeksoo Shin, Kyoung-Joung Lee, Hyo-Sang Lim, Sangwon Hwang, Erdenebayar Urtnasan, Yongmie Jo, Min-Hyuk Kim

**Affiliations:** ^1^Institute for Poverty Alleviation and International Development, Yonsei University Mirae Campus, Wonju, Republic of Korea; ^2^Department of Psychiatry, Yonsei University Wonju College of Medicine, Wonju, Republic of Korea; ^3^Department of Global Public Administration, Yonsei University Mirae Campus, Wonju, Republic of Korea; ^4^Department of Social Welfare, Sangji University, Wonju, Republic of Korea; ^5^Division of Business Administration, Yonsei University Mirae Campus, Wonju, Republic of Korea; ^6^Department of Biomedical Engineering, Yonsei University Mirae Campus, Wonju, Republic of Korea; ^7^Division of Software, Yonsei University Mirae Campus, Wonju, Republic of Korea; ^8^Department of Precision Medicine, Yonsei University Wonju College of Medicine, Wonju, Republic of Korea; ^9^Artificial Intelligence Bigdata Medical Center, Yonsei University Wonju College of Medicine, Wonju, Republic of Korea

**Keywords:** older adults, depression, childhood adversity, stress, social support, moderated mediation, path analysis

## Abstract

**Background:**

As life expectancy increases, understanding the mechanism for late-life depression and finding a crucial moderator becomes more important for mental health in older adults. Childhood adversity increases the risk of clinical depression even in old age. Based on the stress sensitivity theory and stress-buffering effects, stress would be a significant mediator, while social support can be a key moderator in the mediation pathways. However, few studies have tested this moderated mediation model with a sample of older adults. This study aims to reveal the association between childhood adversity and late-life depression in older adults, taking into consideration the effects of stress and social support.

**Methods:**

This study used several path models to analyze the data from 622 elderly participants who were never diagnosed with clinical depression.

**Results:**

We found that childhood adversity increases the odds ratio of depression by approximately 20% in older adults. Path model with mediation demonstrates that stress fully mediates the pathway from childhood adversity to late-life depression. Path model with moderated mediation also illustrates that social support significantly weakens the association between childhood adversity and perceived stress.

**Conclusion:**

This study provides empirical evidence to reveal a more detailed mechanism for late-life depression. Specifically, this study identifies one crucial risk factor and one protective factor, stress and social support, respectively. This brings insight into prevention of late-life depression among those who have experienced childhood adversity.

## 1. Introduction

Childhood adversity, which is also often called adverse childhood experiences (ACEs), refers to children’s exposure to emotional, physical, or sexual abuse, or household dysfunction in a broad sense (1). In this study, we apply a narrower definition of childhood adversity that includes physical, emotional, sexual or general maltreatment that has occurred before the age of 18, following World Health Organization’s definition (2). According to previous research, childhood adversity has been associated with many negative outcomes, both physical and mental, throughout life (3). The short-and long-term consequences of exposure to adversity in childhood are of great importance to public health (4). People experiencing trauma through adverse life events in childhood are at elevated risk of several other psychiatric disorders, including depression, PTSD, behavior problems, substance abuse, self-harm, and suicidal thoughts and attempts (4). Several meta-analyses of cross-sectional and longitudinal studies have consistently reported that people who experienced childhood maltreatment have increased risk of, severity of, and poor treatment of clinical depression (5–9), even in old age (10).

As the average life expectancy has been increasing all over the world, late-life depression has been gaining more and more attention in the academic field. Elderly people are more likely to experience the loss of a spouse or close friends, as well as social isolation, medical comorbidities, neurological disorders. As a result, elderly people have higher risks of clinical depression than any other age groups (11–13).

The pathways by which childhood adversity leads to depression may be mediated in various direct and indirect ways, presenting opportunities for mitigation and intervention strategies (4). However, which mediator and which moderator is present in these pathways remains uncertain (5). A few studies proposed that both biological alterations associated with childhood maltreatment (e.g., alterations in systemic inflammation, the HPA axis, and neural structure and function) (4, 8) and psychosocial factors (e.g., stress, emotional intelligence, attachment, and coping skill) may mediate the increased risk for the development of mood and other disorders (14–17).

According to the stress sensitivity theory, childhood adversity brings early trauma and increases individual stress sensitivity to concurrent life events. People who experience childhood adversity would be exposed to a greater level of stress over their lifetimes, which would increase the risk of late-life depression following stressful life events (18–20).

However, social support could be a significant protective factor to disrupt the link between childhood adversity to late-life depression via perceived stress. It has not fully investigated yet, but so far, some research suggests a positive role of social support as a potential moderator because of its stress-buffering effect in the path from childhood adversity to adult depression (5, 8). One problem is that the study participants of that research were restricted to either young adults or only women. In contrast, our research investigates late-life depression, where previous studies have focused on depression in early adulthood. There have been only a few studies investigating if social support could be a powerful moderator to reduce the link from childhood adversity to late-life depression.

It is uncertain how social support might break the mediation chain from childhood adversity to late-life depression. For example, could social support directly alleviate the causes of late-life depression from childhood adversity? Or, would social support indirectly moderate the pathway from childhood to depression, by moderating the link from childhood adversity to individual stress sensitivity? There is a recent study analyzing the data of elderly people from a community cohort which supported perceived social support as a potential factor for late-life depression (21). Although the authors assumed that the result would be attributable to a stress-buffering effect, a limitation is that the stress level was not measured directly in the study.

Taken together, three hypotheses are tested in this study. Our first hypothesis is that childhood adversity is associated with depression in older adults. So far, many previous studies have demonstrated that childhood adversity increases the risk of depression, but have focused on the outcome in adolescence or young and middle adulthood (9, 22). However, we predict the effects of childhood adversity on depression continue into late adulthood as well. Next, the second hypothesis is that stress mediates the association between childhood adversity and late-life depression. Based on a meta-analysis supporting stress sensitization (18), we anticipate that people who were exposed to a high level of childhood adversity would be more vulnerable to stress in adulthood than those who were not, and that this heightened stress sensitization following childhood adversity would be ultimately associated with a high risk of late-life depression. Finally, the last hypothesis is that social support can alleviate the association between childhood adversity and late-life depression via stress modulation in older adults. A recent study (23) provides empirical evidence suggesting that social support reduces the effects of childhood adversity on neural processing of threat. Although the study sample was restricted to children, we hypothesize that this moderating effect by social support would also be found in late adulthood. By testing this moderation effect, we aim to find a significant intervention factor that could improve mental health in older adults.

## 2. Methods

### 2.1. Participants and procedure

This study analyzed data from 622 participants who had never been diagnosed with clinical depression at a hospital. The original research project was designed to develop a prediction algorithm for late-life depression in a community sample. A sample of 685 elderly participants who were at least 55 years old or older was recruited from the Korean Genome and Epidemiology Study-Cardiovascular Disease Association Study via phone. Participants who agreed to join this research project were invited to the campus for 1 day between December 2020 and April 2021 for baseline data collection. After listening to a detailed explanation of the research project, each participant voluntarily signed the written consent form and entered an interview room to have a 1:1 session with a trained researcher. Trained researchers interviewed each participant with a semi-structured questionnaire and conducted a clinical assessment for approximately 90 minutes. Although the original baseline data includes 685 participants, we excluded the data from 63 participants who had a past medical record of clinical depression, from this study. By controlling for the high relapse rate after the first episode of clinical depression appears, we aimed to avoid overestimating the relationship between childhood adversity and late-life depression. Out of 622 participants who never had any medical records of clinical depression, 23 people were experiencing a current depressive episode while 599 people were not when trained researchers performed the Mini-International Neuropsychiatric Interview (MINI) assessment. More detailed sample characteristics are presented in the Table 1.

**Table 1:** Descriptive statistics.

	Total (*N* = 622)	With depression (*n* = 23)	Without depression (*n* = 599)
Psychological characteristics			
Current depression episode	*N* (%)	*N* (%)	*N* (%)
Yes	23 (3.70%)	23 (100.0%)	–
No	599 (96.30%)	–	599 (100.0%)
Childhood adversity	N (%)	N (%)	N (%)
High (ETI ≥7)	67 (10.77%)	6 (26.09%)	61 (10.18%)
Low (ETI <7)	555 (89.23%)	17 (73.91%)	538 (89.82%)
	M (SD)	M (SD)	M (SD)
Stress	11.95 (4.93)	25.70 (8.29)	11.42 (3.90)
Social support	3.94 (0.69)	2.97 (0.99)	3.98 (0.64)
Medical history			
	*N* (%)	*N* (%)	*N* (%)
High blood pressure (HBP)	276 (44.37%)	12 (52.17%)	264 (44.07%)
Hyperlipidemia (HLP)	241 (38.75%)	17 (73.91%)	224 (37.40%)
Diabetes (DM)	126 (20.26%)	7 (30.43%)	119 (19.87%)
Cardiovascular disease (CVD)	78 (12.54%)	5 (21.74%)	73 (12.19%)
Cerebrovascular disease (CVA)	35 (5.63%)	4 (17.39%)	31 (5.18%)
Cancer (CA)	67 (10.77%)	2 (8.70%)	65 (10.85%)
Demographic characteristics			
Sex	*N* (%)	*N* (%)	*N* (%)
Male	282 (45.34%)	8 (34.78%)	274 (45.74%)
Female	340 (54.66%)	15 (65.22%)	325 (54.26%)
Age	*N* (%)	*N* (%)	*N* (%)
50s	63 (10.13%)	5 (21.74%)	58 (9.68%)
60s	284 (45.66%)	9 (39.13%)	275 (45.91%)
70s	220 (35.37%)	8 (34.78%)	212 (35.39%)
80s	55 (8.84%)	1 (4.35%)	54 (9.02%)
Education	*N* (%)	*N* (%)	*N* (%)
≤Elementary school	172 (27.65%)	5 (21.74%)	167 (27.88%)
Middle or high school	291 (46.78%)	9 (39.13%)	282 (47.08%)
≥2–3 years or 4 years college	159 (25.56%)	9 (39.13%)	150 (25.04%)
	M (SD)	M (SD)	M (SD)
Household income (won; monthly)	3,009,077 (2,238,960)	2,423,478 (1,439,693)	3,032,061 (2,262,316)

ETI, early trauma Inventory.

### 2.2. Measures

#### 2.2.1. Depression

As a screening tool of clinical depression, the MINI assessment (24) was used in this study. We used the Korean version of MINI assessment. Previous research translating the MINI assessment into Korean and testing its validity by 15 psychiatrists with the sample from 10 university hospitals and private clinics (25) demonstrated that the Korean version of MINI assessment has a good concordance with the expert’s diagnoses of major depressive disorder (Kappa = 0.71) and other mental health diseases. When a trained researcher performed this structured test, the test results made a diagnosis out of three choices (a current depressive episode, a past depressive episode, or none).

#### 2.2.2. Childhood adversity

A self-report version of the Early Trauma Inventory (ETI-SF) (26) was used to measure childhood adversity. Previous research testing the reliability and validity of its translated version in Korean (27) demonstrated that the Korean version of ETI-SF has good psychometric properties, exhibiting a comparable construct to that of the original ETI-SF. It includes 30 items in total, but the last 3 items ask more specifically about the most traumatic event among those reported. For this reason, we excluded the final 3 questions from the ETI-SF. We summed up 27 binary items which asked about the presence of specific adverse childhood experiences directly related to general trauma, physical abuse, emotional abuse, or sexual abuse (Cronbach’s alpha = 0.75). In our data, the total ETI scores ranged from 0 to 16. Based on previous research (28), if we recoded it as a binary variable with a threshold of 7 to distinguish the high exposure group from the low exposure group. 10.8% of the sample (*n* = 74) belongs to the high exposure group (ETI-SF ≥7), while 89.2% (*n* = 611) belongs to the low exposure group (ETI-SF < 7).

#### 2.2.3. Stress

For perceived stress, the participants responded to 9 items of the short version of Perceived Stress Inventory (PSI). This self-report measure was developed for the Korean population surveys (29–31) and this short version of PSI was composed of 4 items from Perceived Stress Questionnaire, 4 items from Stress Response Inventory, and 1 item from Stress induced Cognition Scale. The test–retest reliability was ranged from 0.67 to 0.88, which was a satisfactory level. When evaluating its validity using exploratory factor analysis and confirmatory factor analysis with the sample of 387 Korean adults, the short version of PSI showed good reliability and validity (29). Each item was scored from 1 *not at all* to 5 *very true.* Total scores came from summation of the 9 items asking to score the severity of physical and psychological symptoms related to life stress during the last month (Cronbach’s alpha = 0.89).

#### 2.2.4. Social support

Perceived social support was measured by the multidimensional scale of perceived social support (MSPSS) (32). Multiple studies (33–35) have shown that the translated version of MSPSS in Korean demonstrates good concurrent validity and high internal consistency reliability across gender and age groups in Korea. It includes 12 self-report items about social support in everyday life from significant other, family, and friends. The total score was created by summation of the 12 items (Cronbach’s alpha = 0.91).

#### 2.2.5. Covariates

Based on previous research (36, 37), a participant’s age, sex, education, household income, past medical histories were included as covariates. Age was a continuous variable. Sex was a binary variable coded as 0 for a female and 1 for a male. Education was a categorical variable coded as 1 for elementary school degree or less than that, 2 for more than elementary school degree but less than or equal to high school degree, and 3 for equal to or higher than 2–3 years college degree. Household income indicated the average household income in the official currency of South Korea (won). Due to its high skewness, we applied log-transformation for the household income. Past medical histories were measured by six binary items. The six items included whether a participant had experienced high blood pressure, hyperlipidemia, diabetes mellitus, cardiovascular disease, cerebrovascular accident, and cancer in the past.

### 2.3. Statistical analysis

Path models with mediation or moderated mediation were used to investigate the associations from childhood adversity to late-life depression via stress and social support. First, we ran logistic regression models to estimate a crude odds ratio as well as an adjusted odds ratio of childhood adversity for predicting the occurrence of a present depressive episode in older adults. Next, we ran a path model using mediation only to see if a participant’s perceived stress level mediates the association between childhood adversity and later development of clinical depression in older adults. After that, we developed the model to a more complicated path model using moderated mediation. By adding social support as a moderator (W) on the pathway from childhood adversity (an initial predictor, X) to late-life depression (an outcome, Y) as well as on the pathway from childhood adversity to perceived stress (a mediator, M), we examined how perceived social support can ameliorate the negative effects of childhood adversity on late-life depression. In our path analyses, late-life depression (Y) was treated as a categorical variable while stress (M) was treated as a continuous variable. Missing data were handled by multiple imputation with 20 imputations. Among the variables included in our analyses, only household income has some missing data (*n* = 609). STATA 17.0 and Mplus 8.5 were used for the analyses.

## 3. Results

### 3.1. Logistic regression models: crude and adjusted effects of childhood adversity on late-life depression

As shown in Table 2, the logistic regression results predicting depression demonstrated that the crude effect of childhood adversity on late-life depression was statistically significant (crude OR = 1.21, *p* < 0.05). However, this association became non-significant in the adjusted model (adjusted OR = 1.03, *p* = 0.772) after adding other predictors such as stress and social support.

**Table 2:** Logistic regression results: crude and adjusted effects on depression.

Y = Depression		
	Crude OR (95% CI)	Adj. OR (95% CI)
Stress	1.76^***^ (1.59, 1.95)	1.70^***^ (1.51, 1.91)
Childhood adversity	1.21^*^ (1.03, 1.42)	1.03 (0.86, 1.23)
Age	0.97 (0.77, 1.22)	0.94 (0.72, 1.23)
Sex (1 = male)	0.88 (0.70, 1.11)	1.09 (0.82, 1.46)
Education	1.17 (0.93, 1.46)	1.22 (0.96, 1.54)
Income	0.87 (0.70, 1.07)	0.84 (0.65, 1.08)
High blood pressure (HBP)	1.09 (0.87, 1.37)	1.08 (0.84, 1.39)
Hyperlipidemia (HLP)	1.47^***^ (1.20, 1.80)	1.36^*^ (1.07, 1.73)
Diabetes mellitus (DM)	1.13 (0.93, 1.38)	1.10 (0.87, 1.38)
Cardiovascular disease (CVD)	1.13 (0.95, 1.36)	0.99 (0.81, 1.21)
Cerebrovascular accident (CVA)	1.18^*^ (1.03, 1.36)	1.13 (0.95, 1.33)
Cancer (CA)	0.96 (0.75, 1.23)	0.79 (0.58, 1.07)

^*^p < 0.05, ^**^p < 0.01, ^***^p < 0.001.

### 3.2. Path model with mediation only: full mediation from childhood adversity to late-life depression via stress

**Figure 1:**
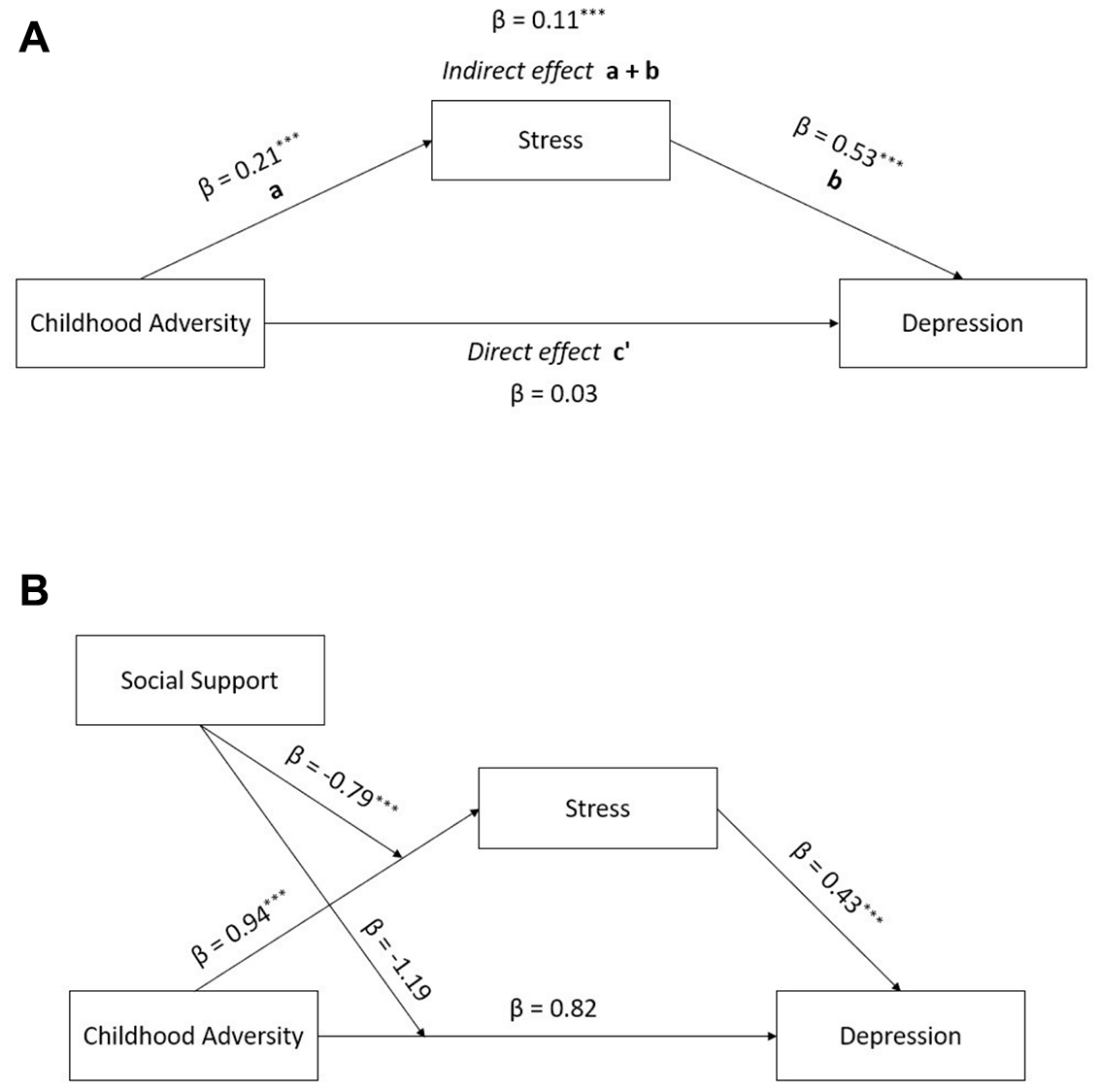
Path results from childhood adversity to depression **(A)** simple mediation model, **(B)** moderated mediation model.

As shown in Figure 1A, the direct path from childhood adversity to depression was not statistically significant (adjusted OR = 0.03, *p* = 0.772). However, the pathway from childhood adversity to stress (adjusted *β* = 0.21, *p* < 0.001) and the pathway from stress to depression (adjusted *β* = 0.53, *p* < 0.001) were significant. Besides separate pathways, this indirect path from childhood adversity to depression via stress was also statistically significant (Adjusted *β* = 0.11, *p* < 0.001) to explain the risk of late-life depression. As a goodness-of-fit measure, *R*^2^ measures indicate that our mediation only model explains 52.3% of the variance of depression and 9.8% of the variance of stress.

### 3.3. Path model with moderated mediation: social support as a significant moderator on the pathway from childhood adversity to stress

**Figure 2:**
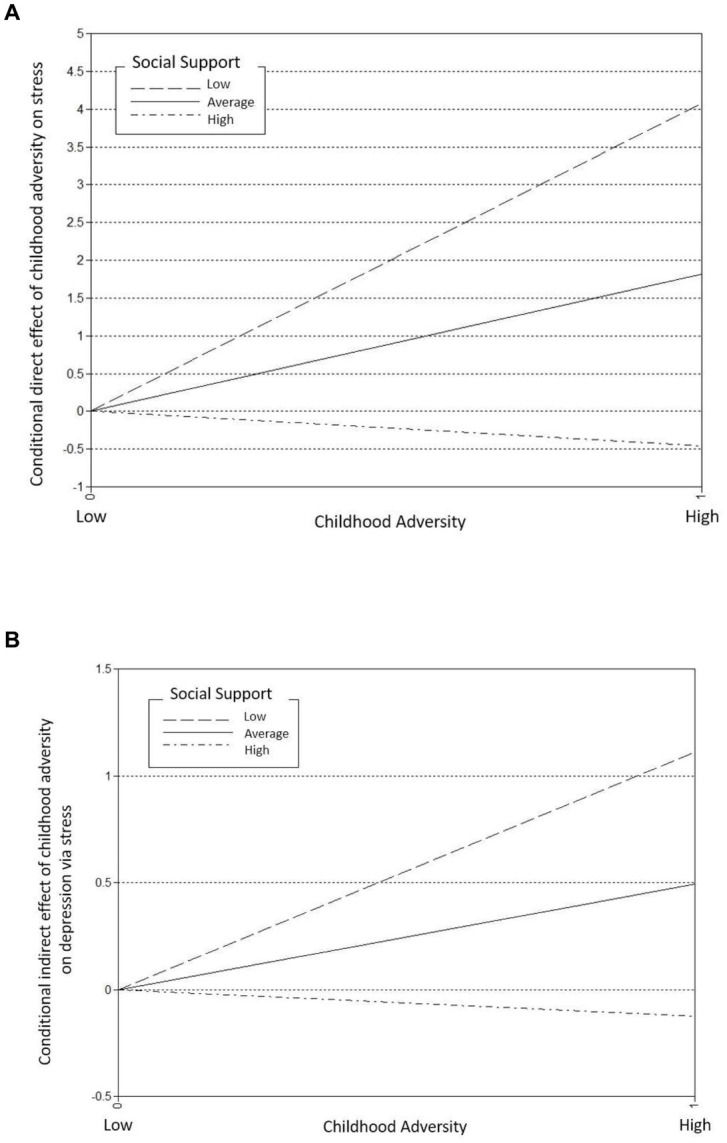
Conditional direct and indirect effects of childhood adversity on stress and depression respectively, moderated by the level of social support **(A)** conditional direct effect of childhood adversity on stress, **(B)** conditional indirect effect of childhood adversity on depression via stress.

As shown in Table 3, social support marginally moderated the direct pathway from childhood adversity to depression (adjusted OR = 0.30, *p* = 0.090) in older adults. However, social support is a significant moderator on the pathway from childhood adversity to stress (adjusted *β* = −0.79, *p* < 0.001). That is, social support alleviates the negative effects of adverse childhood experiences on stress (Figure 1B). Figure 2A displays how the direct effect of childhood adversity on perceived stress differs by the level of perceived social support. As shown in Figure 2A, people who received the low level (M-1SD) or the average level (M) of social support tended to report greater stress if they had been exposed to a high level of adverse childhood experiences. However, the difference by childhood adversity on perceived stress was not found among those who think they received the high level (M + 1SD) of social support (Figure 2A). Furthermore, significant protective effect of social support was observed in the indirect pathway from childhood adversity to late-life depression (Figure 2B). For those who reported the low level (M-1SD) or the average level of social support, childhood adversity was significantly associated with the higher risks of late-life depression. However, when the level of social support was higher than the average (M + 1SD), this association between childhood adversity and late-life depression was not statistically significant any longer (Figure 2B). *R*^2^ measures demonstrate that this moderated mediation model explains 66.7% of the variance of depression and 20.5% of the variance of stress.

**Table 3:** Moderated mediation path model results.

Y = Depression			Y = Stress		
	Crude OR (95% CI)	Adj. OR (95% CI)		Crude *β* (95% CI)	Adj. *β* (95% CI)
Stress	1.76^***^ (1.59, 1.95)	1.53^***^ (1.30, 1.81)	Childhood adversity (A)	0.18^***^ (0.10, 0.25)	0.94^***^ (0.61, 1.27)
Childhood adversity (A)	1.21^*^ (1.03, 1.42)	2.26 (0.84, 6.08)	Social support (B)	−0.08^*^ (−0.16, −0.01)	−0.21^***^ (−0.29, −0.13)
Social support (B)	0.60^***^ (0.53, 0.68)	0.81^*^ (0.68, 0.97)	(A) × (B)	–	−0.79^***^ (−1.12, −0.46)
(A) × (B)	–	0.30 (0.08, 1.21)	Age	−0.30^***^ (−0.37, −0.23)	−0.09^*^ (−0.17, −0.01)
Age	0.97 (0.77, 1.22)	0.91 (0.73, 1.14)	Sex (1 = male)	−0.16^***^ (−0.23, −0.08)	−0.15^***^ (−0.22, −0.07)
Sex (1 = male)	0.88 (0.70, 1.11)	1.12 (0.84, 1.49)	Education	0.07 (−0.46, 0.61)	0.06 (−0.02, 0.14)
Education	1.17 (0.93, 1.46)	1.13 (0.91, 1.41)	Income	−0.05 (−0.13, 0.03)	−0.07 (−0.15, 0.02)
Income	0.87 (0.70, 1.07)	0.85 (0.68, 1.06)	HBP	−0.04 (−0.11, 0.04)	−0.04 (−0.12, 0.04)
HBP	1.09 (0.87, 1.37)	1.10 (0.88, 1.37)	HLP	0.10^*^ (0.02, 0.18)	0.09^*^ (0.01, 0.16)
HLP	1.47^***^ (1.20, 1.80)	1.30^*^ (1.00, 1.69)	DM	−0.07 (−0.15, 0.01)	−0.07 (−0.14, 0.01)
DM	1.13 (0.93, 1.38)	1.10 (0.90, 1.35)	CVD	0.04 (−0.04, 0.12)	0.03 (−0.04, 0.10)
CVD	1.13 (0.95, 1.36)	0.91 (0.75, 1.09)	CVA	0.02 (−0.06, 0.10)	0.01 (−0.06, 0.08)
CVA	1.18^*^ (1.03, 1.36)	1.10 (0.96, 1.27)	CA	0.07 (−0.01, 0.15)	0.08^*^ (0.01, 0.15)
CA	0.96 (0.75, 1.23)	0.94 (0.73, 1.22)			

^*^p < 0.05, ^**^p < 0.01, ^***^p < 0.001. HBP, high blood pressure; HLP, hyperlipidemia; DM, diabetes mellitus; CVD, cardiovascular disease; CVA, cerebrovascular accident; CA, cancer.

## 4. Discussion

This study demonstrates that childhood adversity increases the risk of late-life depression, and this association is fully mediated by individual stress sensitivity. Also, we found that social support moderates the pathway from childhood adversity to depression via alleviating the effects of childhood adversity on stress.

### 4.1. The increased risk of late-life depression by childhood adversity

One of major findings of this study is that childhood adversity increases the risk of late-life depression by approximately 20%. Previous studies report that elderly people with childhood adversity are 2–13 times more likely to have depression compared to those without childhood adversity, although the estimated risks vary depending on the type of or the severity of childhood adversity as well as on the methods used to measure depressive symptoms (e.g., self-report questionnaire or structured interview) (10, 21, 38–41). Here, our target outcome is defined as experiencing a current episode of depression in older adulthood. Generally, those who have been previously diagnosed with clinical depression have a higher occurrence of current depression in late adulthood. To control for this, we excluded participants who had been diagnosed with major depressive disorder at a clinic from our sample. Because of this, our results might underestimate the effect size of childhood adversity in predicting depression. Given that the change in longitudinal effect of childhood adversity on the development of depression over time has not fully explored, further studies are recommended to see if the association between childhood adversity and depression among older adults is static or changes as they become older.

### 4.2. Stress as a mediator on the pathway from childhood adversity to late-life depression

In this research, the major findings support that the negative effects of childhood adversity on late-life depression can be explained by an increased individual stress reactivity. It is in line with many previous empirical studies in neurobiology, genetics, and endocrinology (8, 18–20, 42). According to previous studies, perceived stress can mediate the pathway from childhood adversity to depression (16, 43). Our research strengthens these findings by demonstrating that increased individual vulnerability to stress following childhood adversity persists in old age. This infers that the effects of childhood adversity on depression should be life-long and may even be aggravated over the life course by hyperactivated individual vulnerability to stress. To explain the mechanism of stress as a mediator between childhood adversity and late-life depression, there are two potential hypotheses: One hypothesis is based on the conceptual framework of “stress sensitization.” That is, early-life stress serves as preexisting vulnerability such that exposure to late-life stressors would amplify the risk of depression (43). This is substantiated by neurobiological, genetic, and endocrinological studies (8, 18–20). The other hypothesis is focused on “stress proliferation effect”. According to this hypothesis, initial primary stressors from childhood adversity give rise to additional secondary proliferated stress in different social domains. Since we measured perceived stress using self-report items, it is hard to reveal the role of stress in relation to neurobiological, genetic, and endocrinological studies. As a result, it is not clear which hypothesis would be a more plausible explanation. However, according to a recent study analyzing longitudinal data from the sample of older adults, the hypothesis based on “stress proliferation effect” might make more sense than the hypothesis based on “stress sensitization” (43). With respect to depressive symptoms in older adults, that study also suggests alternative mechanisms by which early-life and later-life stress work together (43). Further study is needed to investigate whether and how the mechanism of individual stress reactivity on the pathway from childhood adversity to depression among older adults differs from that of young adults.

### 4.3. Social support as a moderator of the association between childhood adversity and stress reactivity

This study demonstrates social support can indirectly weaken the association between childhood adversity and depression by alleviating the effects of childhood adversity on perceived stress. Those who perceived high levels of social support among older adults with childhood adversity were likely to report lower levels of perceived stress compared to those who perceived high levels of social support without childhood adversity. The protective effect of social support on the pathway from childhood adversity to stress leads to a significant decrease in the risk of late-life depression.

Our results extend the application of major findings of previous research on older populations. Previous research suggests that social support might directly reduce the risk of depression or moderate the negative effect of childhood adversity on depression (14, 16, 17, 44–47). Although previous research reports the role of social support as a moderator on the pathway from childhood adversity to depression, it does not delineate the mechanism by which social support would moderate the association between childhood adversity and depression in older adults (21). The major findings of our research identify the indirect pathway of how social support moderates the effects of childhood adversity on depression in relation to individual stress reactivity. In psychiatry and psychology fields, social support is hypothesized to protect mental health both directly through the benefits of social relationships and indirectly as a buffer against stressful circumstances (48, 49). However, previous studies often discuss its stress buffering effect without including any direct measure of stress in the data. By revealing the moderated mediation pathway after including the direct measures of perceived stress and perceived social support, we add empirical evidence for the stress buffering effect of social support on depression in older adults.

Of course, though social support could be a protective factor in depression overall, the specific moderation effect by social support in the pathway from childhood adversity to depression might vary over time. For example, Power et al. (44) report that perceived family support does not play a significant role as a buffer in the development of adult depression among those who experienced childhood maltreatment. According to the study (44), it is mainly because childhood maltreatment often occurred within the family. Thus, it may be difficult for survivors to separate their perceived interaction with family members from their experiences of abuse and neglect from family members. It is unclear whether the finding of this tendency can be generalized to older adults, since older adults likely have spent much more time separated from original family members following marriage with a new partner and developing new family relationships with their children and grandchildren. It would be interesting for further research to explore this dynamic in the moderation effect of social support over time.

One interesting insight of this research is that social support should be considered as a crucial element in the prevention of depression in older adults with childhood adversity. Further studies are recommended to identify which sources of social support would be more effective (e.g., support from family vs. support from friends) and if different strategies are warranted by gender. Moreover, it would also be informative to explore the biological impacts of social support. For example, some studies reported that social support from close relationships moderate the effect of child adversity in neural responses or HPA axis of children and adolescents (23, 50). The question of whether these findings could be applicable to older adults remains unanswered.

### 4.4. Limitations of the study

Several limitations should be considered when interpreting our results. First, this study analyzed cross-sectional data. Of course, it does not mean that our testing relationships among key variables are unreliable because the time point for each measure was obviously different. Specifically, the items measuring childhood adversity asked about participants’ childhood experiences until the age of 18. The items measuring social support indicated the average level of support from family and friends during the last year, while the items measuring stress asked perceived stress symptom severity during the past month. And MINI measured the participants’ mental health statuses at that moment data was collected from them. Since each variable reflects the participants’ experiences during specific moments in time, we adjust for the common criticism of cross-sectional designs, that the mediation path can be constructed out of order. But due to the possibility that self-report retrospective measures can bring recall bias on participants’ responses here, future study is recommended to design a longitudinal prospective study to account for potential impact of depressive episodes on responses if their budget allows. Our research fails to control that participants experiencing a current episode of depression may have distorted cognition, which can make reports of childhood adversity or currently perceived stress more negative than would otherwise be the case. Second, even after the participants who had been previously diagnosed with clinical depression were excluded from the study sample to minimize the recall bias of the people with a depression episode in the past, there were several participants who never visited a clinic and did not have any medical records of depression despite indications they had a past episode of clinical depression from our MINI interview. Future research should differentiate these participants from the sample, and we will investigate if findings are similar in the older adults who never had any episode of clinical depression compared to those who did have a past episode but never visited a clinic. By doing this, we expect to obtain a clearer understanding of if childhood adversity would increase the risk of new onset late-life depression. Next, we dichotomized the sample based on the cut-off value suggested by a recently published article that investigated a global sample. When we used the continuous ETI variable, the results for both the mediation path and the moderated mediation path were similar to those obtained with the dichotomized ETI variable. For this research, we chose to use the dichotomized ETI variable because we believed that comparing a high exposure group with another group would provide a clearer view of how social support moderates the mediation path from traumatic events to stress and to depression, rather than estimating point values through a unit increase of one point per traumatic event. Since there is limited research specifically investigating the cut-off value of the ETI variable for the Korean sample, and we did not anticipate that the effects of exposure to childhood adversity on depression in the Korean sample would differ from those in the global sample, we adopted the cut-off value from a previous study analyzing a global sample. Further research is recommended to determine the universality of this cut-off value. Lastly, due to small sample sizes, we were not able to perform subgroup analyses by the severity or the type of childhood adversity. Future research analyzing a larger sample is recommended to include subgroup analyses.

## 5. Conclusion

Despite the limitations discussed above, this study contributes empirical evidence to support the association between childhood adversity and late-life depression in older adults. In particular, this study reveals the full mediation pathway via perceived stress that explains the association between childhood adversity and late-life depression. Also, this study sheds light on the moderation role of social support, indicated by a moderated mediation path model. By alleviating the effect of childhood adversity on individual stress sensitivity, social support can serve as a crucial protective factor to reduce the risks of late-life depression.

## Data availability statement

The datasets for this article are not publicly available due to concerns regarding participant/patient anonymity. Requests to access the datasets should be directed to the corresponding author.

## Ethics statement

The studies involving human participants were reviewed and approved by Yonsei Mirae Campus Institutional Review Board (1041849-202212-SB-223-09). The patients/participants provided their written informed consent to participate in this study.

## Author contributions

M-HK initiated this study and supervised the development of the manuscript. J-kL contributed to the data cleaning as well as the methods and results sections of the manuscript. JL contributed to the introduction and discussion sections of the manuscript. M-KC is the principal investigator of the overall research project. JP and TS contributed to developing the semi-structured questionnaire used in this study. K-JL, H-SL, SH, and EU contributes to developing a prediction algorithm for late-life depression using wearable devices. YJ managed the data collection process at the baseline survey. All authors contributed to the article and approved the submitted version.

## Funding

This study was supported by the Ministry of Education of the Republic of Korea and the National Research Foundation of Korea (NRF-2020S1A5A2A03045088).

## Conflict of interest

The authors declare that the research was conducted in the absence of any commercial or financial relationships that could be construed as a potential conflict of interest.

## Publisher’s note

All claims expressed in this article are solely those of the authors and do not necessarily represent those of their affiliated organizations, or those of the publisher, the editors and the reviewers. Any product that may be evaluated in this article, or claim that may be made by its manufacturer, is not guaranteed or endorsed by the publisher.

## References

[1] Felitti VJ Anda RF Nordenberg D Williamson DF Spitz AM Edwards V. Relationship of childhood abuse and household dysfunction to many of the leading causes of death in adults: the adverse childhood experiences (ACE) study. Am J Prev Med. (1998) 14:245–58. doi: 10.1016/S0749-3797(98)00017-8, PMID: 9635069

[2] WHO. Preventing child maltreatment: a guide to taking action and generating evidence World Health Organization (2006). Available at: https://apps.who.int/iris/handle/10665/43499

[3] Ege MA Messias E Thapa PB Krain LP. Adverse childhood experiences and geriatric depression: results from the 2010 BRFSS. Am J Geriatr Psychiatry. (2015) 23:110–4. doi: 10.1016/j.jagp.2014.08.014, PMID: 25306195 PMC4267899

[4] Nelson CA Scott RD Bhutta ZA Harris NB Danese A Samara M. Adversity in childhood is linked to mental and physical health throughout life. BMJ. (2020) 371:m3048. doi: 10.1136/bmj.m3048, PMID: 33115717 PMC7592151

[5] Braithwaite EC O’Connor RM Degli-Esposti M Luke N Bowes L. Modifiable predictors of depression following childhood maltreatment: a systematic review and meta-analysis. Transl Psychiatry. (2017) 7:e1162. doi: 10.1038/tp.2017.140, PMID: 28675390 PMC5538120

[6] Infurna MR Reichl C Parzer P Schimmenti A Bifulco A Kaess M. Associations between depression and specific childhood experiences of abuse and neglect: a meta-analysis. J Affect Disord. (2016) 190:47–55. doi: 10.1016/j.jad.2015.09.006, PMID: 26480211

[7] Lindert J von Ehrenstein OS Grashow R Gal G Braehler E Weisskopf MG. Sexual and physical abuse in childhood is associated with depression and anxiety over the life course: systematic review and meta-analysis. Int J Public Health. (2014) 59:359–72. doi: 10.1007/s00038-013-0519-5, PMID: 24122075

[8] Lippard ETC Nemeroff CB. The devastating clinical consequences of child abuse and neglect: increased disease vulnerability and poor treatment response in mood disorders. Am J Psychiatry. (2020) 177:20–36. doi: 10.1176/appi.ajp.2019.19010020, PMID: 31537091 PMC6939135

[9] Nanni V Uher R Danese A. Childhood maltreatment predicts unfavorable course of illness and treatment outcome in depression: a meta-analysis. Am J Psychiatry. (2012) 169:141–51. doi: 10.1176/appi.ajp.2011.11020335, PMID: 22420036

[10] Wang Y Chen X Zhou K Zhang H. A meta-analysis of the effects of childhood maltreatment on elderly depression. Trauma Violence Abuse. (2022) 24:1593–607. doi: 10.1177/15248380211073838 35232293

[11] Alexopoulos GS. Depression in the elderly. Lancet. (2005) 365:1961–70. doi: 10.1016/S0140-6736(05)66665-2, PMID: 15936426

[12] Mitchell AJ Subramaniam H. Prognosis of depression in old age compared to middle age: a systematic review of comparative studies. Am J Psychiatry. (2005) 162:1588–601. doi: 10.1176/appi.ajp.162.9.1588, PMID: 16135616

[13] Taylor WD. Depression in the elderly. N Engl J Med. (2014) 371:1228–36. doi: 10.1056/NEJMcp1402180, PMID: 25251617

[14] Struck N Krug A Feldmann M Yuksel D Stein F Schmitt S. Attachment and social support mediate the association between childhood maltreatment and depressive symptoms. J Affect Disord. (2020) 273:310–7. doi: 10.1016/j.jad.2020.04.041, PMID: 32421618

[15] Su Y D’Arcy C Meng X. Social support and positive coping skills as mediators buffering the impact of childhood maltreatment on psychological distress and positive mental health in adulthood: analysis of a national population-based sample. Am J Epidemiol. (2020) 189:394–402. doi: 10.1093/aje/kwz275, PMID: 31907548

[16] Vranceanu AM Hobfoll SE Johnson RJ. Child multi-type maltreatment and associated depression and PTSD symptoms: the role of social support and stress. Child Abuse Negl. (2007) 31:71–84. doi: 10.1016/j.chiabu.2006.04.010, PMID: 17215039 PMC1839899

[17] Zhao J Peng X Chao X Xiang Y. Childhood maltreatment influences mental symptoms: the mediating roles of emotional intelligence and social support. Front Psychiatry. (2019) 10:415. doi: 10.3389/fpsyt.2019.00415 31316399 PMC6611427

[18] Bunea IM Szentagotai-Tatar A Miu AC. Early-life adversity and cortisol response to social stress: a meta-analysis. Transl Psychiatry. (2017) 7:1274. doi: 10.1038/s41398-017-0032-3 29225338 PMC5802499

[19] Hammen C Henry R Daley SE. Depression and sensitization to stressors among young women as a function of childhood adversity. J Consult Clin Psychol. (2000) 68:782–7. doi: 10.1037/0022-006X.68.5.782, PMID: 11068964

[20] McLaughlin KA Conron KJ Koenen KC Gilman SE. Childhood adversity, adult stressful life events, and risk of past-year psychiatric disorder: a test of the stress sensitization hypothesis in a population-based sample of adults. Psychol Med. (2010) 40:1647–58. doi: 10.1017/S0033291709992121, PMID: 20018126 PMC2891275

[21] Cheong EV Sinnott C Dahly D Kearney PM. Adverse childhood experiences (ACEs) and later-life depression: perceived social support as a potential protective factor. BMJ Open. (2017) 7:e013228. doi: 10.1136/bmjopen-2016-013228, PMID: 28864684 PMC5588961

[22] LeMoult J Humphreys KL Tracy A Hoffmeister J-A Ip E Gotlib IH. Meta-analysis: exposure to early life stress and risk for depression in childhood and adolescence. J Am Acad Child Adolesc Psychiatry. (2020) 59:842–55. doi: 10.1016/j.jaac.2019.10.011 31676392 PMC11826385

[23] Wymbs NF Orr C Albaugh MD Althoff RR O’Loughlin K Holbrook H. Social supports moderate the effects of child adversity on neural correlates of threat processing. Child Abuse Negl. (2020) 102:104413. doi: 10.1016/j.chiabu.2020.104413, PMID: 32065988 PMC8060780

[24] Sheehan DV Lecrubier Y Sheehan KH Amorim P Janavs J Weiller E. The Mini-International Neuropsychiatric Interview (M.I.N.I.): the development and validation of a structured diagnostic psychiatric interview for DSM-IV and ICD-10. J Clin Psychiatry. (1998) 59:22–33. 9881538

[25] Yoo S-W Kim Y-S Noh J-S Oh K-S Kim C-H Nam Koong K. Validity of Korean version of the Mini-international neuropsychiatric interview. Anxiety Mood. (2006) 2:50–5.

[26] Bremner JD Bolus R Mayer EA. Psychometric properties of the early trauma inventory–self report. J Nerv Ment Dis. (2007) 195:211–8. doi: 10.1097/01.nmd.0000243824.84651.6c, PMID: 17468680 PMC3229091

[27] Jeon J-R Lee E-H Lee S-W Jeong E-g Kim J-H Lee D. The early trauma inventory self report-short form: psychometric properties of the Korean version. Psychiatry Investig. (2012) 9:229. doi: 10.4306/pi.2012.9.3.229, PMID: 22993521 PMC3440471

[28] Almuwaqqat Z Wittbrodt M Young A Lima BB Hammadah M Garcia M. Association of early-life trauma and risk of adverse cardiovascular outcomes in young and middle-aged individuals with a history of myocardial infarction. JAMA Cardiol. (2020) 6:336–40. doi: 10.1001/jamacardio.2020.5749 PMC7666433 33185652

[29] Lee ES Shin HC Lee JH Yang YJ Cho JJ Ahn G. Development of the perceived stress inventory: a new questionnaire for Korean population surveys. Korean J Fam Med. (2015) 36:286. doi: 10.4082/kjfm.2015.36.6.286, PMID: 26634094 PMC4666863

[30] Lee E Shin H Yang Y Cho J Ahn K Kim S. Development of the stress questionnaire for KNHANES: report of scientific study service Korea Disease Control and Prevention Agency (2010).

[31] Shin HC. Measuring stress with questionnaires. J Korean Med Assoc. (2013) 56:485–95. doi: 10.5124/jkma.2013.56.6.485, PMID: 37266941

[32] Wilcox S. Multidimensional scale of perceived social support. Psychol Trauma. (2010) 2:175–82.

[33] Park H Nguyen T Park H. Validation of multidimensional scale of perceived social support in middle-aged Korean women with diabetes. Asia Pac J Soc Work Dev. (2012) 22:202–13. doi: 10.1080/02185385.2012.691719

[34] Kim M Yeom H-E Jung MS. Validation and psychometric properties of the multidimensional scale of perceived social support among Korean breast cancer survivors. Asia Pac J Oncol Nurs. (2022) 9:229–35. doi: 10.1016/j.apjon.2022.01.004, PMID: 35571625 PMC9096736

[35] Park G Hwang Y Kim JH Lee DH. Validation of the South Korean adolescents version of the multidimensional scale of perceived social support. Psychol Sch. (2022) 59:2345–58. doi: 10.1002/pits.22613

[36] Akhtar-Danesh N Landeen J. Relation between depression and sociodemographic factors. Int J Ment Health Syst. (2007) 1:e4. doi: 10.1186/1752-4458-1-4, PMID: 18271976 PMC2241832

[37] DeJean D Giacomini M Vanstone M Brundisini F. Patient experiences of depression and anxiety with chronic disease: a systematic review and qualitative meta-synthesis. Ont Health Technol Assess Ser. (2013) 13:1–33. PMID: 24228079 PMC3817854

[38] Comijs HC Beekman AT Smit F Bremmer M van Tilburg T Deeg DJ. Childhood adversity, recent life events and depression in late life. J Affect Disord. (2007) 103:243–6. doi: 10.1016/j.jad.2007.01.012, PMID: 17291592

[39] Comijs HC van Exel E van der Mast RC Paauw A Oude Voshaar R Stek ML. Childhood abuse in late-life depression. J Affect Disord. (2013) 147:241–6. doi: 10.1016/j.jad.2012.11.010, PMID: 23196199

[40] Raposo SM Mackenzie CS Henriksen CA Afifi TO. Time does not heal all wounds: older adults who experienced childhood adversities have higher odds of mood, anxiety, and personality disorders. Am J Geriatr Psychiatry. (2014) 22:1241–50. doi: 10.1016/j.jagp.2013.04.009, PMID: 24012227

[41] Ritchie K Jaussent I Stewart R Dupuy AM Courtet P Ancelin ML. Association of adverse childhood environment and 5-HTTLPR genotype with late-life depression. J Clin Psychiatry. (2009) 70:1281–8. doi: 10.4088/JCP.08m04510, PMID: 19573496 PMC3078522

[42] Zhong X Ming Q Dong D Sun X Cheng C Xiong G. Childhood maltreatment experience influences neural response to psychosocial stress in adults: an fMRI study. Front Psychol. (2019) 10:2961. doi: 10.3389/fpsyg.2019.02961, PMID: 31993010 PMC6971063

[43] Arpawong TE Mekli K Lee J Phillips DF Gatz M Prescott CA. A longitudinal study shows stress proliferation effects from early childhood adversity and recent stress on risk for depressive symptoms among older adults. Aging Ment Health. (2022) 26:870–80. doi: 10.1080/13607863.2021.1904379, PMID: 33784211 PMC8673399

[44] Powers A Ressler KJ Bradley RG. The protective role of friendship on the effects of childhood abuse and depression. Depress Anxiety. (2009) 26:46–53. doi: 10.1002/da.20534, PMID: 18972449 PMC2629811

[45] Salazar AM Keller TE Courtney ME. Understanding social support’s role in the relationship between maltreatment and depression in youth with foster care experience. Child Maltreat. (2011) 16:102–13. doi: 10.1177/1077559511402985, PMID: 21471145 PMC4099379

[46] Sperry DM Widom CS. Child abuse and neglect, social support, and psychopathology in adulthood: a prospective investigation. Child Abuse Negl. (2013) 37:415–25. doi: 10.1016/j.chiabu.2013.02.006, PMID: 23562083 PMC3672352

[47] Su Y Meng X Yang G D’Arcy C. The relationship between childhood maltreatment and mental health problems: coping strategies and social support act as mediators. BMC Psychiatry. (2022) 22:359. doi: 10.1186/s12888-022-04001-2 35619058 PMC9137127

[48] Cohen S Wills TA. Stress, social support, and the buffering hypothesis. Psychol Bull. (1985) 98:310–57. doi: 10.1037/0033-2909.98.2.310, PMID: 3901065

[49] Gariepy G Honkaniemi H Quesnel-Vallee A. Social support and protection from depression: systematic review of current findings in Western countries. Br J Psychiatry. (2016) 209:284–93. doi: 10.1192/bjp.bp.115.169094, PMID: 27445355

[50] Hostinar CE Gunnar MR. Social support can buffer against stress and shape brain activity. AJOB Neurosci. (2015) 6:34–42. doi: 10.1080/21507740.2015.1047054, PMID: 26478822 PMC4607089

