# Supplementary material for: Childhood adversity and late-life depression: moderated mediation model of stress and social support
Source: Front Psychiatry. 2023 Jun 26;14:1183884. doi: 10.3389/fpsyt.2023.1183884 (PMC10331618; doi:10.3389/fpsyt.2023.1183884)
Supplement: Supplementary file 1 [file Table_1.docx]

Supplement Table. Correlations

|  | Dep | ACE | Str | SS | Age | Sex | Edu | Inc | HBP | HLP | DM | CVD | CVA | CA |
| --- | --- | --- | --- | --- | --- | --- | --- | --- | --- | --- | --- | --- | --- | --- |
| Dep | 1.00 |  |  |  |  |  |  |  |  |  |  |  |  |  |
| ACE | 0.10^*^ | 1.00 |  |  |  |  |  |  |  |  |  |  |  |  |
| Str | 0.55^***^ | 0.18^***^ | 1.00 |  |  |  |  |  |  |  |  |  |  |  |
| SS | -0.28^***^ | -0.14^***^ | -0.30^***^ | 1.00 |  |  |  |  |  |  |  |  |  |  |
| Age | -0.01 | -0.03 | -0.08^*^ | -0.10^**^ | 1.00 |  |  |  |  |  |  |  |  |  |
| Sex | -0.04 | 0.16^***^ | -0.16^***^ | -0.04 | 0.22^***^ | 1.00 |  |  |  |  |  |  |  |  |
| Edu | 0.05 | -0.03 | 0.01 | 0.07 | -0.16^***^ | 0.14^***^ | 1.00 |  |  |  |  |  |  |  |
| Inc | -0.05 | 0.01 | -0.04 | 0.10^*^ | -0.39^***^ | 0.02 | 0.40^***^ | 1.00 |  |  |  |  |  |  |
| HBP | 0.03 | 0.09^*^ | -0.04 | -0.08 | 0.25^***^ | 0.15^***^ | -0.10^*^ | -0.08 | 1.00 |  |  |  |  |  |
| HLP | 0.14^***^ | 0.00 | 0.10^*^ | -0.07 | 0.13^**^ | -0.17^***^ | -0.13^**^ | -0.07 | 0.25^***^ | 1.00 |  |  |  |  |
| DM | 0.05 | -0.01 | -0.07 | -0.04 | 0.12^**^ | 0.09^*^ | -0.11^**^ | -0.12^**^ | 0.21^***^ | 0.16^***^ | 1.00 |  |  |  |
| CVD | 0.05 | 0.01 | 0.04 | -0.11^**^ | 0.16^***^ | 0.06 | -0.08^*^ | -0.08 | 0.14^***^ | 0.10^*^ | 0.16^***^ | 1.00 |  |  |
| CVA | 0.10^*^ | 0.03 | 0.02 | -0.06 | 0.17^***^ | 0.02 | -0.05 | -0.08^*^ | 0.13^***^ | 0.05 | 0.09^*^ | 0.16^***^ | 1.00 |  |
| CA | -0.01 | -0.02 | 0.07 | -0.01 | 0.08 | 0.04 | -0.03 | -0.11^**^ | 0.03 | -0.01 | 0.04 | 0.04 | 0.05 | 1.00 |

^*^*p* <.05, ^**^*p* <.01, ^***^*p* <.001.

*Note.* Dep = depression, ACE = childhood adversity, Str = stress, SS = social support, Age = age, Sex = sex (1 = male, 0 = female), Edu = education (1 = equal to or less than elementary school degree, 2 = higher than elementary school degree and equal to/less than high school degree, 3 = equal to or higher than 2-3 year college degree), Inc = average monthly household income, HBP = high blood pressure, HLP = hyperlipidemia, DM = diabetes mellitus, CVD = cardiovascular disease, CVA = cerebrovascular accident, CA = cancer.
